# Situation of Occupational and Environmental Health in Mexico

**DOI:** 10.29024/aogh.2317

**Published:** 2018-08-31

**Authors:** Martha Edilia Palacios Nava, Ana Rosa Moreno Sánchez, María del Pilar Paz Román, Juan José García García, Rodolfo Nava Hernández

**Affiliations:** 1 Public Health Department, Faculty of Medicine, National Autonomous University of Mexico, MX; 2 Occupational Health, Graduate Studies Division, Faculty of Medicine, MX

## Abstract

**Background:**

Mexico has a great diversity and richness of natural resources, but evaluations of the quality of life of Mexicans show the deep inequalities and the gap between rich and poor. While 5% of families concentrate 58% of the wealth, the health spending in environment and health is 0.2 and 2.7 of the GDP respectively. This has repercussions both on the gradual deterioration of the environment and on the insufficient health and social security coverage of the working population.

**Objective:**

To describe the current situation of occupational and environmental health in Mexico.

**Methods:**

A bibliographic review was performed on the socioeconomic, demographic, environmental, legal and health status of the Economically Active Population (EAP).

**Findings:**

There is a constant deterioration of terrestrial and marine ecosystems, accompanied by an increase in environmental pollution in large cities. The unemployment rate of the EAP has decreased in one year to 3.4%, but the informal labor rate reached 57.3%, which translates into population without social security. Compliance with legislation for the protection of workers’ health is insufficient. The recent amendments to the law have meant a setback in these respects. The reported information on accidents and occupational diseases corresponds to only 34% of workers. There has been a decrease in the rate of work accidents in the last six years, but an increase in diseases and permanent disabilities. During 2016, the first cause of occupational illness was hearing loss, but the profile was dominated by musculoskeletal diseases, which together reached 36.5%.

**Conclusions:**

To improve the occupational and environmental health situation, it is necessary to implement general and particular measures against inequalities, increase the budget in health and environment, enforce legislation and expand social security coverage to the population. These measures should be part of public policies as well as actions of academics and researchers.

## Introduction

The nexus that people have with work, inside or outside of it, has an important influence on health [1]. This is evidenced, on the one hand, in the general morbidity and mortality profiles of the population, in which the increase in cardiovascular diseases, diabetes, cancer and obesity could be related to factors and demands of work [2] and on the other, in registered accidents and occupational diseases. In the same way, the damage to health that can occur in those who do not have a job or those who have it but in precarious conditions is important [3].

The damage to health produced by or related to work and the lack of access to health and social security services are aspects related to the lack of equity. “In this regard it should be noted that the Commission of Social Determinants for Health of the World Health Organization (WHO) has established that work, in particular, under appropriate and decent employment conditions can, in fact, complement the needs that allow to reduce inequities in health in its most broad concept” [3].

In the same way, various elements of social organizations, such as the distribution of wealth and national and local power, explain most of the health inequities. These depend on the adopted policies [4]. Therefore, inequities in health are the product of social inequities [5].

According to this approach, this article addresses the situation of occupational and environmental health in Mexico. The objective was to describe, with a global perspective, the socioeconomic context of the country, the unequal and inequitable distribution of resources, their manifestation in the environment and the lack of coverage for health care and social security of the working population.

### Methods

A literature review was carried out. The document was structured in three parts that go from general to specific.

The first part describes the overview of the country, its population and the socioeconomic context. For this, texts and national and international reports that allowed contrast the inequality and inequity in the distribution of resources were selected.

In the second section, documents were selected to show an overview of environmental deterioration, the lack of compliance with public policies and some examples of the damage to health investigated.

The third part describes the occupational health situation. It defines those who make up the working population, the legislation that should protect them, the institutions in charge of providing social security and/or attention to their health and inadequacies in coverage. The main damages in productive age in general and accidents and work-related diseases in particular are also described. It is pertinent to specify that there are no comprehensive diagnoses in occupational health area so for the description, articles, books and reports of the main institutions responsible for addressing the administrative, legal and health aspects of the working population were used. Information on work accidents and illnesses was obtained from the only social security institution that reports data on work risks and serves the largest proportion of the workforce in the country.

## Overview of the Country

Mexico has a great diversity and richness of natural resources [6], oil among them. The country had a revolution in the early twentieth century, which generated a new constitution that established, among other things, the obligations of employers and the rights of the working population. Three decades later, the Federal Labor Law and the Social Security Law had already been promulgated. In spite of this, the country, like other developing countries, has a great insufficiency, both in the application of the current legislation and preventive measures, as well as in the attention to health problems generated by work and the environment.

**Figure 1:**
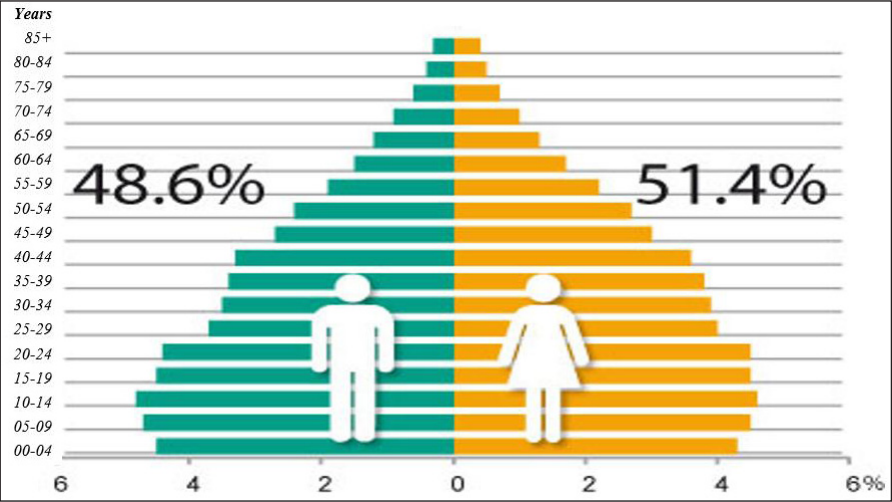
Population Pyramid Mexico 2015.

According to the latest census, the population amounted to 119.5 million persons. Population dynamics have changed in recent years, showing a slowing of growth; there has been an increase in the proportion of people between 25 and 64 years of age, which constitute 65% of the population. The group of 65 years or more represents 7.2%; nevertheless, it is the one that has had the greatest growth, so the general population is in the process of aging (Figure 1) [7]. The population under 15 years old represents 27% of the total; 76.8% of the population is urban. Life expectancy at birth is 75.2 years, 78 in women and 73 in men, fertility rate is 2.2, gross mortality rate is 5.8, and infant mortality rate is 11.7% [7].

Currently, the epidemiological pattern shows that the main causes of death are chronic non-communicable diseases, such as diabetes, ischemic heart diseases, stroke, cancer and liver cirrhosis, which occur mainly in people over 40 years of age [7].

Mexico is a member of the Organization for Economic Cooperation and Development (OECD). The growth of the Gross Domestic Product (GDP) in 2015 and 2016 was 2.5 and 2.2 respectively [8]. The country’s macro-economy has withstood the consequences of falling oil prices. It had also been possible to control inflation until this year, in which, due to increasing gas prices, inflation soared to 6.66%, the highest in 16 years [9].

Evaluations of the quality of life of Mexicans, performed by various national and international organizations, show the deep inequalities and the gap between rich and poor. The OECD mentions that, with a low social spending, poverty and inequality remain high the country has the lowest performance in security and corruption, as well as in education. Likewise, it occupies the last place of the OECD countries, with 2.7% of GDP in health spending [8], because, in spite of the gradual growth in life expectancy, the country has one of the last places in the health situation of the member countries of this organization [10].

Inequality is also evident in what was reported by the United Nations Development Program (UNDP); the index that evaluates the distribution of income and human development in the different regions of the country was 0.587, down from countries with similar economies, such as Chile and Argentina [11].

Regarding internal evaluations, the National Council for the Evaluation of Social Development (Consejo Nacional Para la Evaluación del Desarrollo Social – CONEVAL) [12] reported that 43.6% of the population is in poverty and 7.6% in extreme poverty. Similarly, there is a lack of social security in 55.8%, health services in 15.5%, and food in 20.1%.

One of the most important problems is insecurity, since the homicide rate increased to 21.6 per 100,000 inhabitants in the last year, which means that it is 14 times higher than in East Asia and the Pacific [11].

## Environmental Health

In 2002, the Federal Commission for the Protection of Health Risks (Comisión Federal para la Protección de Riesgos Sanitarios – COFEPRIS) of the Ministry of Health prepared the “First National Diagnostic of Environmental and Occupational Health. In the same year, he prepared the action program: Environmental Health, 2001–2006. This proposal incorporated the lines of action for Strengthening Environmental Health and Improvement of Occupational Health [13].”

In the 2007–2012 program, the action priorities were “clean beaches, atmospheric pollutants, indoor exposure to wood smoke, physicochemical and bacteriological quality of water for human use and consumption and climate change [14].”

In the 2013–2018 health program, actions are generally planned to prevent controlling and monitoring the harmful effects of environmental factors, occupational hazards, import and export of products and services, advertising and international health [15].

Although the Ministry of Health has carried out programs and actions, these have been insufficient to stop the constant deterioration of the environment, the scarce investment and lack of compliance with regulations is manifested in the constant increase in environmental problems.

Among them is that 73% of water bodies are contaminated; 80% of discharges from urban centers and 85% of industrial discharges are dumped directly into them without previous treatment [16]. This creates severe impacts on human health and on ecosystems. The depletion of groundwater, especially in the north of the country, promotes natural contamination by arsenic and fluorine in northern and central regions; this adds to oil pollution in oil areas and problems related to flooding during the hurricane seasons.

Chemical contamination generates chronic exposures at low doses, whose effects are associated with an increase in cancer in areas contaminated with arsenic [17,18]. From the hydrographical basins, 33% present a strong water pressure, determined mainly by the human settlements and the agricultural activity. More than half the population in the country, 53%, lives in these basins, mainly in the north and center of Mexico [16].

The increase in the production of solid waste in the country has been presented in an important way in the rural and urban areas in the last decades. In 2011, around 41 million tons of urban solid waste were generated, equivalent to almost 112,500 tons/day. Only between 2003 and 2011, the total generation of these wastes increased by 25%. These wastes have a significant impact on ecosystems, both through air and water. Metropolitan areas accounted for 43% of the total waste [19].

Outdoor air pollution occurs in the main cities of the country, as well as in industrial and petrochemical development zones. It is an environmental risk that causes not only diseases and hospitalizations but also deaths, due to acute and chronic exposures.

Regarding the metropolitan area of Mexico City, if tougher measures were implemented for PM10 contamination, around 2,300 deaths per year could be reduced. In the case of ozone, it would be possible to avoid about 400 deaths, especially in those over 65 years of age [20].

A postmortem study of 21 young persons in Mexico City found that the heart begins to show the adverse effects of air pollution at an early age and that the problem may worsen in the presence of small pieces of inactivated bacteria that attract pollutants [21]. Millions of urban children are chronically exposed to high concentrations of atmospheric pollutants, i.e. fine particles (PM2.5) and ozone, which exposure is associated with an increased risk of asthma, lung cancer, heart disease and Alzheimer’s disease. Compared with children living with clean air, one study showed that those in Mexico City exhibit systemic, cerebral and intrathecal inflammation, attention and short-term memory deficits, damage to the endothelial and epithelial barriers, neural autoantibodies, Alzheimer’s and Parkinson’s symptoms [22].

It is estimated that 25 million people in the country living in rural areas use solid fuels (mainly firewood) as their main source of domestic energy [23]. In these intramural environments, combustion is usually carried out in open heaters, where the highest levels of air pollution in the country are reached; PM2.5 levels can reach up to 1,000 μg/m^3^ [24].

The predominant sources of persistent organic compounds (POC) have been agriculture, power generation, industry and poor waste management. Mexico participates with Latin American countries in the development of a monitoring system that includes surveillance of POC in human biological matrices (blood and milk) to verify the progressive reduction of exposure to these substances [25].

Regarding metals, lead remains one of the main concerns for populations that are exposed either by the place where they live, or where non-ferrous metallurgical activities are performed, as well as by mining where gold and silver are extracted. There are more than 800 new exploration and mining projects in the country, which may eventually damage the health of the population [14]. Another topic of interest continues to be the elaboration of glazed earthenware by which poor and indigenous populations are exposed to both occupational and peri-occupational pollution. Other metals of importance in the deterioration of public health are mercury, manganese and, to a lesser extent, cadmium and chromium.

The presence of other pollutants in Mexico, such as hydrocarbons and pesticides, continues to be a cause for concern, due to both their effects on the environment and health risks. According to the Federal Office for Environmental Protection (Procuraduría Federal de Protección Ambiental – PROFEPA) [26], in 2015, 423 inspection visits were performed at companies where soil damage was detected due to contamination by hazardous materials and waste. Every year, environmental emergencies associated with oil and its derivatives (diesel, fuel oil, gasoline), LP gas, natural gas and agrochemicals occur [14].

With regard to acute pesticide poisoning, between 2001–2010 the annual average was 3,928 cases nationwide. The main reported causes were lack of regulations, deficiencies in educational and risk information [27], as well as the inadequate disposition of the containers and the storage of agrochemicals, and the lack of participation in decision making. This remains one of the most important occupational health problems in rural areas due to the lack of control in the use of pesticides, the high social vulnerability of farmers, their poverty, lack of education and health care, and problems with land tenure, in addition to environmental problems, such as climate change [27].

According to one study, in rural areas, the poorest and most marginalized population is the one formed by migrants, agricultural migrant male and female laborers, the latter being the most disadvantaged. The authors report in laborers from Sinaloa a prevalence of anemia 5.6 times higher than in men and double to that detected in the national health survey for the rural population. In addition, they were six times more likely to become ill with asthma, twice as parasitic, twice the respiratory and stomach illnesses, and 38% more heart disease [28].

The living conditions of this and other populations increase health risks; it is estimated that approximately four million children, mainly in developing countries, die annually from causes associated with both pre-transitional environmental factors (lack of sanitary conditions, intramural air pollution, etc.) and emerging conditions (persistent chemical compounds, plasticizers, etc.) [14].

In the same way, the rise of temperatures and heat waves associated with climate change can affect food security through the impact of heat on farmers’ productivity. This phenomenon also impacts construction workers, street vendors, peasants, and policemen, among others. They do not have rest (break) times, shaded conditions to protect themselves against ultraviolet radiation, or availability of sufficient drinking water to withstand the heat wave [29].

## Occupational Health

### Economically Active Population

In Mexico the Economically Active Population (EAP) includes a population of 15 or more years of age who are working or looking for work. For the first quarter of 2017 the Economically Active Population (EAP) increased to 53.7 million, which corresponds to 59.2% of the total [30]. From the total of EAP, 62% are men and 38% are women; 96.6 were employed [30]. Regarding the sector of economic activity, 12.6% work in the primary sector, 25.5% in the secondary sector, and 61.4% in the service sector [30].

Although unemployment went from 4% to 3.4% between 2016 and 2017, labor informality increased to 57.3%. This includes those workers who, although working for formal economic units, do so in ways that avoid registration for social security. All of them totaled 29.7 million people, up 1.8% compared to 2016. This aspect is very important for statistical information on accidents and occupational diseases, since only social security institutions have coverage for work risks and only one of them publishes them [30].

#### Child labor

Children are not considered economically active population. This implies that, like informal workers, they do not have health or social security services.

Mexico initiated the systematic recording of child labor in 2007 with the application of surveys. Since then, the survey has not covered migrant children living in collective shelters in agricultural fields, making it difficult to quantify the number of boys, girls and adolescents (children of working migrant agricultural laborers) who work. It also does not include domestic work, or other types of street work, such as “watching over cars,” cleaning windshields, singers [31], etc.

**Figure 2:**
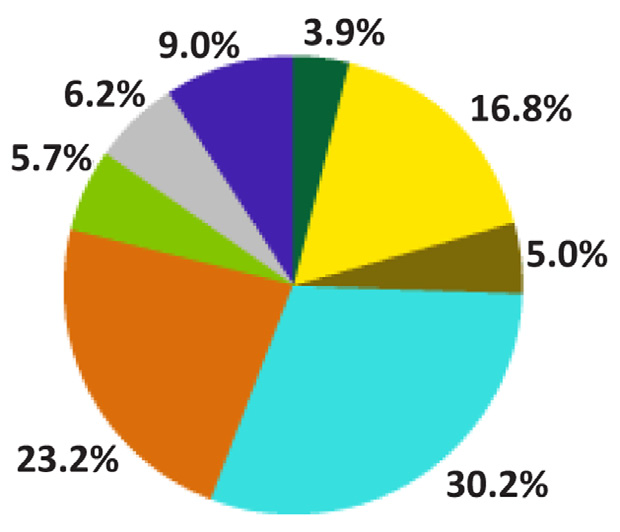
Working population 5 to 17 years of age, according to the job they do. National Survey of Employment, Mexico 2015.

In 2015, there were 2,475,989 children and adolescents from five to 17 years old who did some economic activity; of these, 90% had non-permitted occupations, of which 60% were considered dangerous occupations. Seventy percent were children and 30% were girls. Of the total, 42.5% received no income and 28.8% received up to a minimum wage [32] (approximately $136 per month). In terms of occupations, 30.2% worked in agricultural, forestry, hunting or fishing activities; 23.2% in mining, construction and industry; 16.8% in trade and sales; and the rest in other activities [33] (Figure 2).

### Occupational Health Legislation and Institutions Responsible for Compliance

#### Legal framework of workers’ health

The Mexican Constitution is the supreme law of the country. In it, Article 123 establishes the right to a decent and socially useful work. It specifies the characteristics of what should be working conditions, workers’ rights, and employers’ obligations. It also includes two large sections that define the classification of workers, their employment relationship, and the law that will rule them [34].

Section A includes the regulations regarding workers, day laborers, domestic workers, craftsmen, and, in general, any contract of employment in the private initiative. In this section, sections XIV and XV specify the responsibilities and obligations of employers with respect to accidents and occupational diseases of workers. The Federal Labor Law, which is the second most important law, is derived from this section. Section B establishes the employment relationship between the powers of the union and its workers, which is specified in the Federal Law on Workers Serving the State. From the Federal Labor Law derives the Federal Occupational Safety and Health Regulations, and from this the Official Mexican Standards [34].

### Federal Labor Law

From its first issue, in 1931, the Federal Labor Law included in its title IX everything corresponding to “work hazards”. This law, valid until 2012, could be considered “advanced”, protective of the rights of workers’ health, and a model followed by several countries in Latin America. Its content was progressive, the problem was its low compliance [35].

It was not until 2012 when, as part of the structural reforms of the current government, the federal law had important modifications, among them outsourcing, hourly work, and the change of the table of valuation of permanent disabilities produced by labor risks to an annex [34].

Regarding specific aspects related to health, hygiene, safety, and occupational hazards, the current version of the mentioned law maintains, as in previous versions, Title IX, Article 473, which defines the hazards of work, considered as the accidents and illnesses to which the workers are exposed in performing or because of the same work. In other articles of this same section, the law includes the types and payments for incapacity and compensation for work hazards and other aspects related to their prevention and medical care of workers inside and outside the work centers.

Article 513 contains the list of 161 work-related diseases, which are grouped in different types: 30 different types of pneumoconiosis and brocopulmonary diseases due to different exposures and work processes; 17 diseases of the respiratory tract produced by inhalation of gases and vapors; 18 skin diseases; 16 occupational ophthalmopathies; 36 intoxications; 21 infections, parasites, mycoses and viral infections; two diseases caused by contact with biological products; 10 diseases caused by mechanical factors and variations of the natural elements of the working environment; one disease caused by ionizing and electromagnetic radiation (except cancer); four types of cancer; and six endogenus diseases (hearing loss, cramps, laryngitis, etc.) [35].

In February this year, the Chamber of Deputies approved an amendment to the table of work diseases. In the review, 49 diseases were eliminated and 73 were added, leaving the table with 185 occupational diseases. Among the most important changes are the recognition of diseases associated with the exposure to psychosocial and ergonomic risk factors, as well as the increase in the recognized number of cancers of work origin, which increased from four to 23, and infectious and parasitic diseases, which increased from 21 to 40 [36,37]. Those modifications to the law have not yet been published in the official journal of the federation.

### Health Illness of the Economically Active Population

The Ministry of Work and Social Prevention (Secretaría del Trabajo y Previsión Social – STPS) is in charge of establishing and monitoring compliance with the legislation, as well as coordinating the job of professionals and institutions involved in the health of workers. Attention to illness and social security of the workers are the responsibility of the Ministry of Health and the different Institutions of Social Security.

In Mexico there is no universal system of coverage for health care. The National Health System (Secretaría de Salud – SSA) concentrates all the organizations, institutions, and resources, and together they cover 83% of the population. Access to health services is conditioned by the labor or socioeconomic situation of the Mexican population. Attention to the population not insured for their employment is given by the Ministry of Health (hospitals, clinics, and health centers of the State).

The National Institute of Social Security (Instituto Mexicano del Seguro Social – IMSS) serves workers in the private sector and decentralized services and their dependents (section A of the Constitution), including health care, maternity coverage, old age disability, retirement, death and a well-structured insurance of work hazards. Its financing is tripartite (government, employers, and workers). This institution is the only one that reports accident statistics and work-related diseases in the country, and it covers 32.9% of the total population [38].

The Institute of Security and Social Services for the Workers of the State (Instituto de Seguridad y Servicios Sociales de losTrabajadores del Estado – ISSSTE) has the same social security services as the IMSS and a limited service of work hazards and work-related medical issues. It is financed by the government and the workers, covering 7.4% of the population [38].

Other services that include social security coverage are the Instituto de Social Security for the Armed Forces (Seguridad Social para las Fuerzas Armadas – ISSFAM) and Mexican Oil (Petróleos Mexicanos – PEMEX), covering .007% of the population [38].

Finally, the Popular Insurance (Seguro Popular) provides health services for those who are not affiliated with social security, covers a package of health actions and the fee is defined according to the economic situation of the families, although in some places local governments have assumed the costs. This coverage reaches 43.5% of the population [38].

#### Health Disease in productive age

The overall mortality rate in men and women considered to be of productive age (15–64 years) was 286.5 × 100,000; the three main causes of death in this group correspond to malignant tumors, diabetes, and ischemic heart disease. However, in men it reached almost 400 × 100,000, almost double that of women. This is mainly due to the increase of violent deaths in the 15–29 and the 30–44 age groups, in which the main causes were homicides, traffic accidents, and suicides. In these same groups, there is an increase in ischemic heart disease, renal failure, cirrhosis, diabetes, and breast cancer [10,39].

Other health problems in people over 20 years of age are reported by the national health and nutrition survey. In this study, a prevalence of diabetes of 9.4% was found, which increased to 10.3 in women. Hypertension was 25.5% and overweight and obesity combined had a prevalence of 72.5% (37% overweight and 38.6% obesity) [38].

#### Accidents and work-related diseases

As previously mentioned, the IMSS is the only institution that reports information on work-related accidents and diseases, which are considered by law as work hazards. The total number of workers affiliated during 2016 was 18,206,112 [40]. In the same year, the economically active population increased to 54,200,000 [41], therefore the statistical data would correspond to 33.6% of the PEA. The frequency of accidents and work-related illnesses of 66.4% of men and women of productive age in the country is unknown.

When analyzing the evolution of work hazards (diseases, accidents at work and on the way to/from work) in the last six years, we observed that the rate was 2.9 × 100 workers during 2016, 1.2% less than in 2011 [40] (Table 1).

**Table 1:** Evolution of accidents, occupational diseases and their severity Mexico 2011–2016.

Rate	2011	2012	2013	2014	2015	2016

Total accidents and diseases*	3.6	3.6	3.3	3.3	3.1	2.9
Work accidents*	2.8	2.8	2.6	2.4	2.4	2.2
Accidents on the way*	0.7	0.8	0.7	0.7	0.6	0.7
Work diseases**	2.74	3.09	3.92	4.93	6.84	6.93
Permanent disabilities***	50.2	49.2	53.1	53.9	59.4	60.9
Lethality**	2.94	2.76	2.42	2.51	2.62	2.66

* × 100 workers; ** ×10,000 workers; *** × 1,000 workers. Own preparation with data source of the statistical memory of IMSSS 2016.

Transportation accidents to/from work remained the same during the period; work accidents dropped from 2.8% to 2.2%, but diseases increased from 3 to 6.9 per 10,000 workers [40] (Table 1).

In the same period, the lethality of labor risks gradually decreased from 2011 to 2014, but as of this year there was an increase. While permanent disabilities have maintained an increase between 2011 and 2016, they grew from 50.2 to 60.9 × 1000, mainly in work accidents [40] (Table 1).

There was a minimal difference in the rate of accidents and illnesses between men and women: 3 and 2.8 respectively. The highest rates in men were in the younger age groups, while in the latter these were found in older age groups (Table 2).

**Table 2:** Incidence of work risks by sex and age Instituto Mexicano del Seguro Social Mexico 2016.

Age groups	Hazards of works per every 100 workers		

	Men	Women	Total

	3.0	2.8	2.9
15–19	3.2	2.0	2.7
20–24	4.0	2.8	3.6
25–29	3.3	2.6	3.0
30–34	2.9	2.6	2.8
35–39	2.7	2.7	2.7
40–44	2.6	2.9	2.7
45–49	2.5	3.1	2.7
50–54	2.5	3.4	2.8
55–59	2.6	3.4	2.8
60–64	2.8	3.0	2.9
65–69	2.0	2.2	2.1
70–74	1.6	1.4	1.5
75+	0.9	0.7	0.9

Statistical memory of IMSS 2016.

According to the economic activity group, occupational accidents had the highest rates in self-service stores and specialized departments, 4.5%; food, beverage, and tobacco sales, 3.5%; and food and beverage preparation and service of temporary accommodation, 3.4%; the incidence was not far from the average rate. However, the most lethal accidents occurred in the economic groups of land transport, construction of buildings and civil engineering works, and purchase of raw materials, materials and auxiliaries, with 96, 56 and 31 deaths per 10,000 accidents respectively [40] (Table 3).

**Table 3:** Groups of economic activity with higher rates of work accidents, permanent disabilities and death Mexico, 2016.

		Work accidents × 100 workers	Permanent disabilities × 1,000 workers	Lethality × 10,000 accidents

1.	Purchase and sale in self-service stores and specialized departments by merchandise line	4.5	0.6	2.4
2.	Food sales, beverages and tobacco products	3.5	0.9	19.4
3.	Preparation and service of food and beverages	3.4	0.6	5.4
4.	Temporary accommodation services	3.4	0.5	2.5
5.	Manufacture of metal products, except machinery and equipment	3.3.	2.4	**22.5**
6.	Food processing	3.0	1.4	**16.7**
7.	Construction of buildings and civil engineering works	2.9	1.7	**56.1**
8.	Ground transportation	2.7	1.7	**95.7**
9.	Purchase of raw materials, materials and auxiliaries	2.6	1.2	**31.0**
10.	Manufacture of rubber and plastic products	2.6	1.5	9.4

Own preparation with data source of the statistical memory of IMSS 2016.

In contrast, the incidence of work-related diseases, according to the economic activity group, showed a great difference when compared to the national average, so the basic metal industry had 98 diseases × 10,000 workers, the extraction and benefit of metallic minerals: 84.6, and temporary accommodation services: 14.6 × 10,000. The highest frequency of permanent disabilities and deaths occurred in the first two groups. The lethality was also high in agriculture [40] (Table 4).

**Table 4:** Groups of economic activity with higher rates of work-related diseases, permanent disabilities and deaths Mexico 2016.

		Work diseases × 10,000 workers	Permanent disabilities × 1,000 diseases	Lethality × 10,000 diseases

1.	Basic metal industries	98.0	11.5	10.0
2.	Extraction and benefit of metallic minerals	84.6	12.7	33.9
3.	Temporary accommodation services	14.6	0.1	0.0
4.	Construction, reconstruction and assembly of transport equipment and its parts	11.8	0.8	0.0
5.	Manufacture of metal products, except machinery and equipment	10.2	1.0	0.0
6.	Manufacture of clothing and other articles based on textiles and various materials, except footwear	10.2	0.5	0.0
7.	Ground transportation	8.8	0.8	0.0
8.	Manufacture and/or assembly of machinery, equipment, appliances, accessories and electrical and electronic articles and their parts	8.0	0.3	0.0
9.	Farming	7.5	0.1	29.8
10.	Purchase and sale in self-service stores and specialized departments by merchandise line	6.0	0.1	0.0

Own preparation with data source of the statistical memory of IMSS 2016.

Regarding occupational diseases, between 2011 and 2016 there was an increase in the general incidence. Reports of the incidence rate of each disease are not published; there are only absolute numbers and percentages with respect to the total of those registered (Table 5).

**Table 5:** Comparison of number and type of work-related diseases registered in 2011 and 2016.

Work-related disease		Number 2011	Percentage	Number 2016	Percentage

Total		4,105	100.0	12,622	100.0
1.	Hearing loss	1,341	32.7	1,873	14.8
2.	Dorsopathies	124	3.0	1,663	13.2
3.	Diseases of the eyes and surroundings	35	0.9	1,364	10.8
4.	Pneumoconiosis	792	19.3	1,017	8.1
5.	Intoxications	33	0.8	876	6.9
6.	Other enthesopathies	123	0.3	700	5.5
7.	Carpal tunnel syndrome	147	3.6	636	5.0
8.	Dermatitis by contact	123	3.0	580	4.6
9.	Injuries in the shoulder	110	2.7	503	4.0
10.	Radial styloid tenosynovitis (from Quervain)	140	3.4	422	3.3
11.	Respiratory conditions due to inhalation of fumes, gases, vapors and chemicals	239	5.8	378	3.0
12.	Other synovitis, tenosynovitis and bursitis	144	3.5	349	2.8
13.	Infectious and parasitic diseases	42	1.0	229	1.8
14.	Epicondylitis	44	1.1	184	1.5
15.	Mental and behavioral syndromes	31	0.8	168	1.3
16.	Osteoarthritis	25	0.6	150	1.2
17.	Decompression sickness	2	0.0	108	0.9
18.	Peripheral vascular disease	22	0.5	76	0.6
19.	Occupational cancer	3	0.1	35	0.3
20.	Asthma	11	0.3	30	0.2
21.	Several of lower frequency	574	14.0	1,281	10.1

Statistical memory of IMSS 2016.

**Figure 3:**
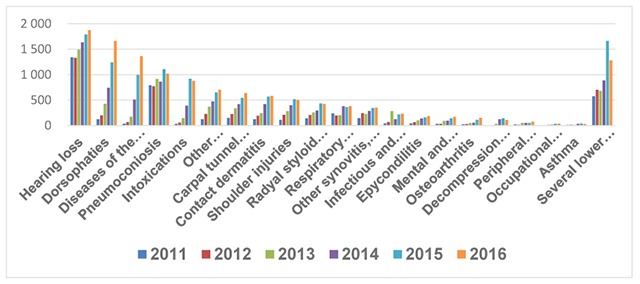
Number of occupational diseases IMSS 2011–2016.

Hearing loss has always been at the top of the list, but its percentage has declined from 32.7% to 14.8%, mainly due to the increase in diseases of the musculoskeletal system. From 2011 to 2016 there has been a constant increase of dorsopathies, diseases of the eye, enthesopathy, carpal tunnel syndrome, and pneumoconiosis [40] (Figure 3). During 2016, although the first cause of illness was hearing loss, the profile was dominated by musculoskeletal diseases, which together reached 36.5% (Table 5).

#### Human resources

In Mexico, 52% of medical schools teach Medicine or Health at Work in the undergraduate program; [42] in addition, the National Autonomous University of Mexico (Universidad Nacional Autónoma de México – UNAM) has a social service program in occupational health. The IMSS and PEMEX, with the academic validity from the UNAM, have the specialty of Occupational and Environment Medicine, with medical residency modality: the same university and four other universities have a specialization program in occupational health without medical residency [43]. In an institute and nine public universities, there is a master program in Occupational Health; two of these universities have a doctoral program. There are also short courses related to the area, given by public and private universities [44]. According to the last publication on the topic, in 2006, 846 specialists had graduated, from which 720 were active, implying “one specialist for every 21,006 workers [43],” when “an advisable ratio of one per 5,000 is estimated [43].” To date, according to the postgraduate information on Occupational Health of the UNAM, 1385 specialists have graduated [44]. Of the specialists, only 255 doctors are certified [45]. Nevertheless, about 13,000 physicians, of whom their training is unknown, work as company doctors [44].

## Conclusions

Mexico has great wealth and has experienced a slow but steady growth [5]. However, different evaluations performed by national and international organizations show inequality in the country [5,7,8]. While 5% of the wealthiest families concentrate 58% of the wealth [46], the country, along with Bolivia, Guatemala, and Panama, has experienced a decline in the purchasing power of wages. Investment in health and environment, with 2.7 and 0.2 of GDP, is among the lowest in Latin America [46]. All this determines the current situation of the occupational and environmental health and the living and working conditions of the population. For more than 15 years, the ministries in charge of health and the environment have carried out programs that propose actions to improve the situation. However, due to insufficient budget and poor compliance with current regulations, there has been an accelerated deterioration of terrestrial and marine ecosystems, together with a constant increase in rural and urban pollution. Only in 2014 an ecological disaster occurred in Sonora, when a mining incident spilled 40,000 m^3^ of copper sulphate, sulfuric acid, and metals in a river [47].

In the area of occupational health, unemployment, compared to other countries, such as Argentina or Spain, is low [48,49]. However, there is inequality due to the high rate of informal work (57.3%) [30]. This generates precarious work, without social security, economic benefits, or access to health services.

The amendments to the law have contributed to this situation with the flexibility in hiring, the legalization of outsourcing, and the consequent increase in the absence of fringe benefits [34].

At now, the working conditions in Mexico are something officially unknown, since there is no registration or evaluation. But the tragedies that have occurred show the lack of compliance with regulations. This is the case of what happened in a coal mine in 2006, where 65 miners died due to violation of hygiene and safety standards [50]. This is also what happened in a clandestine sewing workshop in which, during the September 19th earthquake, an unknown number of workers were buried [51].

Regarding accidents and occupational diseases, it is necessary to consider that the reported corresponds approximately to 34% of the total EAP; thus, what happens with the rest of the workers is unknown. To this, we would have to add the under-registration for different circumstances. In the case of accidents, one study showed that, on average, 26.3% (range 0–68%) was not rated as such [52]. As to occupational diseases, a report from the IMSS [53] itself, estimated an under-registry of 92%; if this were so, we would be talking about an occurrence of almost 157,775 work diseases and a rate of 86.66 × 10000, very similar to the one that is recorded in the extractive industry [40].

In addition to accidents and diseases classified as work-related, we must take into account the presence of diseases in productive age, which may be related to work too, such as cardiovascular disease, diabetes, obesity, and chronic kidney disease, among others, in which there has been an association with certain working conditions [2].

To improve working conditions and reduce accidents and diseases, it is important that what is stipulated in the law is fulfilled, but it is equally important that workers have social security, both for their care and to really know the scale of the problem. It is also necessary to have more professionals in occupational and environmental health, carry out quantitative assessments that allow mapping of risk factors and damage to health, and train workers to prevent accidents and work-related illnesses.

According to the International Labor Organization (ILO), in order to reduce the growing number of occupational diseases, it is essential to implement preventive measures [54]. The same principle should be applied to the general morbidity and mortality of the population in general and of the population of productive age in particular, since, in the end, work and the environment have an impact on the health of the entire population.

However, these measures would have to go beyond the scope of secondary prevention and consider the social determination of health [4], which implies that in order to eliminate health inequity, it is necessary to take action against inequalities: “improving living conditions, combating the distribution inequality of power, money and resources, to measure the magnitude of the problem, to analyze it, and to evaluate the effects of the interventions [55].” The latter could be achieved, both with the information generated by the recently created National Observatory of Inequities of Health [50,51,52,53], and with the participation of academics, physicians, and social researchers who contribute to this purpose, documenting and disseminating the current situation in their own fields.

For the rest of recommendations, it is necessary that the public policies are proposed and implemented following a principle of social justice.
